# A bibliometric analysis of the top 50 most-cited articles in glaucoma management

**DOI:** 10.1097/MD.0000000000050617

**Published:** 2026-09-11

**Authors:** Beta Abuhadi, Naya F. Alzahrani, Fai H. Alotaibi, Jumanah M. Aldirbas, Fatema M. Almulhim, Jumana I. Alhashmi Alamir, Lina Justenia, Najla A. Alsubaie, Razan A. Abuhekmah, Fatima A. Almadhloum Alsuwaidi, Abdulwahab M. Alqaffas, Rawan A. Abuhekmah, Fahad AlHarthi

**Affiliations:** a College of Medicine, King Abdulaziz University, Jeddah, Saudi Arabia; b Medical Student, College of Medicine, King Abdulaziz University, Jeddah, Saudi Arabia; c College of Medicine, Almaarefa University, Riyadh, Saudi Arabia; d Medical Student, College of Medicine, Almaarefa University, Riyadh, Saudi Arabia; e College of Medicine, Jouf University, Sakaka, Saudi Arabia; f Medical Student, College of Medicine, Jouf University, Sakaka, Saudi Arabia; g College of Medicine, King Faisal University, Al-Ahsa, Saudi Arabia; h Medical Student, College of Medicine, King Faisal University, Al-Ahsa, Saudi Arabia; i College of Medicine, King Saud University for Health Sciences, Jeddah, Saudi Arabia; j Medical Student, College of Medicine, King Saud University for Health Sciences, Jeddah, Saudi Arabia; k King Abdullah International Medical Research Center, Jeddah, Saudi Arabia; l Medical Student, King Abdullah International Medical Research Center, Jeddah, Saudi Arabia; m College of Medicine, University of Jeddah, Jeddah, Saudi Arabia; n Medical Student, College of Medicine, University of Jeddah, Jeddah, Saudi Arabia; o College of Medicine, University of Sharjah, Sharjah, United Arab Emirates; p Medical Student, College of Medicine, University of Sharjah, Sharjah, United Arab Emirates; q Glaucoma Division, King Khaled Eye Specialist Hospital, Riyadh, Saudi Arabia.

**Keywords:** bibliometric analysis, glaucoma, glaucoma management, surgical management

## Abstract

**Background:**

As the foremost cause of irreversible vision loss around the world, glaucoma continues to be the leading factor behind irreversible blindness. Even with progress in diagnostic tools, surgical techniques, and additional therapies, the disease continues to be problematic because of its asymptomatic onset and the partial effectiveness of pressure-reducing treatments. The overall role of significant publications in guiding glaucoma research has not been thoroughly evaluated.

**Methods:**

A bibliometric study was carried out to determine the 50 articles on glaucoma with the highest citation counts, with searches conducted in the Web of Science database. Four reviewers independently screened the articles. The extracted information covered authorship, country of origin, journal name, study design, level of evidence, citation counts, and thematic focus. Citation and co-occurrence mapping were applied.

**Results:**

The 50 most-cited articles accrued 4054 citations (mean: 81.1), peaking in the 2000s (42%). The US (52%) and UK (30%) led contributions, primarily in Ophthalmology and British Journal of Ophthalmology. Level II evidence predominated (44%), followed by Level IV evidence (38%). Retrospective cohort studies comprised the most common study design (36%), followed by case series (30%), while randomized controlled trials represented 12% of the included studies. Key themes included diagnostic integration, trabeculectomy, minimally invasive surgery (MIGS), and refractory disease management.

**Conclusion:**

Most-cited glaucoma-management literature was characterized by retrospective study designs and representation of both traditional filtration surgery and emerging device-based approaches, including MIGS. These findings describe patterns of scholarly attention within highly cited glaucoma-management literature and should not be interpreted as trends across the entire field or as evidence of comparative clinical effectiveness.

## 1. Introduction

Glaucoma, often referred to as the “slow and silent thief of sight,”^[1]^ affects more than vision alone, impacting patient independence, mental health, and overall quality of life.^[1]^ It remains a major global health concern, affecting more than 76 million people worldwide, with this number projected to exceed 111 million by 2040, predominantly among the elderly.^[2]^ As the leading cause of irreversible blindness, glaucoma continues to represent a significant public health challenge.

Elevated intraocular pressure (IOP) remains the most important modifiable risk factor. However, lower IOP is associated with reduced visual-field progression.^[3]^ While IOP reduction through medical or surgical approaches, including trabeculectomy and minimally invasive glaucoma surgery (MIGS), remains the cornerstone of treatment, these limitations highlight the importance of continued advances in glaucoma diagnosis and surgical management.^[4]^

Advances in diagnostic imaging, particularly optical coherence tomography, have improved the early detection of glaucomatous damage.^[5]^ Nevertheless, challenges persist in identifying pre-perimetric disease and detecting subtle progression over time,^[5]^ underscoring the need for more sensitive and specific diagnostic approaches.

Given these challenges, a comprehensive analysis of the literature is warranted. Examining the Top 50 most-cited articles in glaucoma management provides insight into key scientific contributions, research trends, and existing knowledge gaps. Although bibliometric studies can describe broad patterns in ophthalmic research, the present study examines a clinically oriented corpus of the 50 most-cited articles identified through a glaucoma-management search strategy. It characterizes citation patterns, study designs, levels of evidence, and thematic clusters, with particular attention to diagnostic assessment, surgical interventions, and management of complications. By focusing on highly cited literature relevant to these clinical domains, this study provides a focused description of scholarly attention in glaucoma care and identifies areas for future research.

## 2. Methodology

### 2.1. Search strategy

A comprehensive search of the Web of Science Core Collection was conducted on July 5, 2025, to identify the most-cited articles related to glaucoma. The search was performed in the Web of Science Core Collection because of its standardized citation indexing, reproducible citation ranking, and compatibility with bibliometric analyses. The title-field query was TI = (glaucoma) AND TI = (management). No document type filter or publication-year restriction was applied to capture influential glaucoma publications across the full historical development of the field. Only English-language publications primarily focused on glaucoma were included. All retrieved studies were ranked according to total citation counts, and articles with higher citation numbers were prioritized during the selection process. No ethical approval was required because the study analyzed previously published bibliographic data and did not involve human participants, patient data, or animal subjects.

### 2.2. Study selection

**Figure 1.**
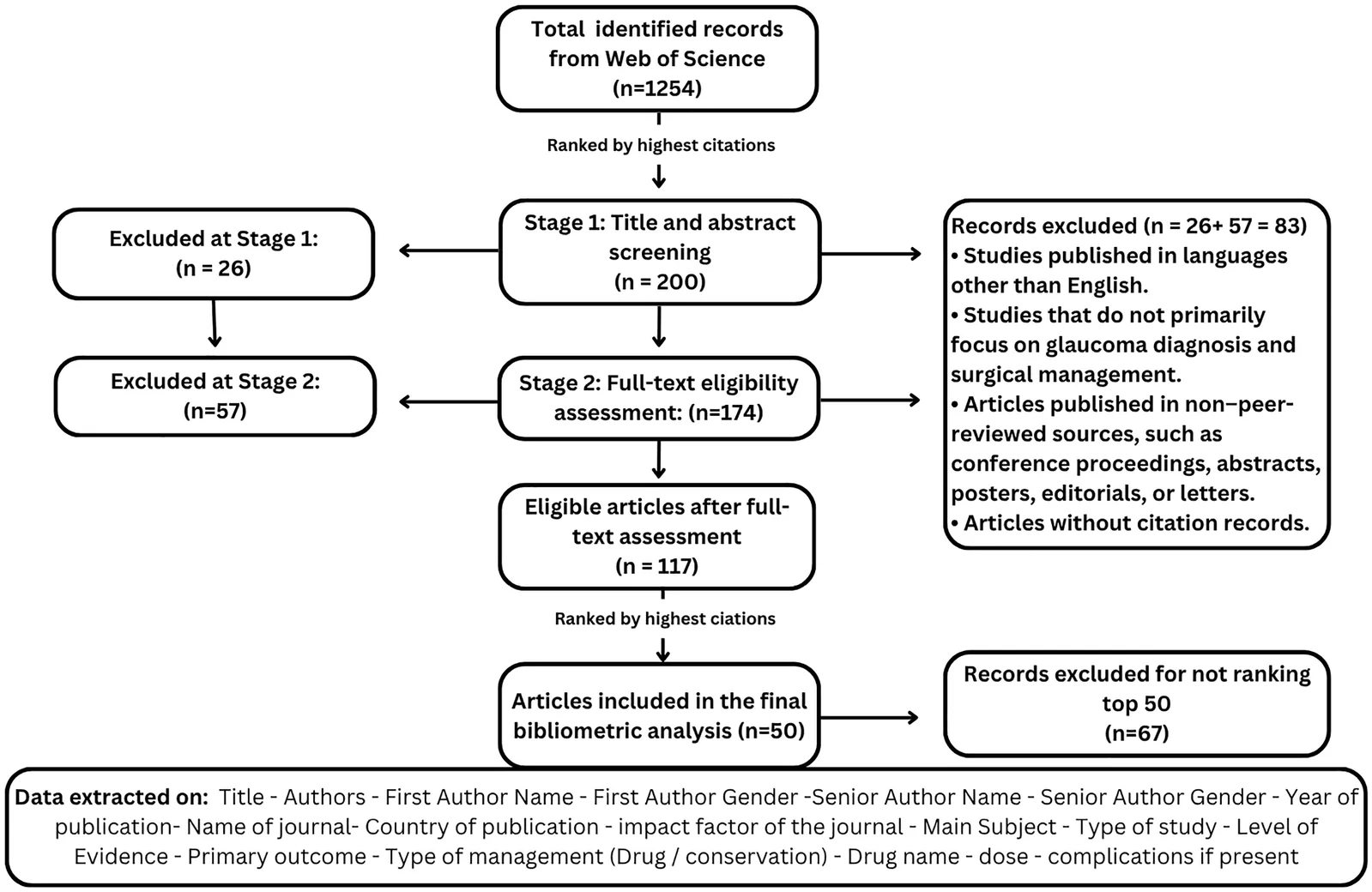
Flowchart of the literature search and study selection process for the Top 50 most-cited glaucoma articles.

The literature was independently screened by 4 reviewers. Titles and abstracts were assessed according to predefined eligibility criteria. Studies were included if they were indexed in the Web of Science Core Collection, published in English, primarily focused on glaucoma, and had received at least 1 citation. No publication-year restriction was applied. Studies were excluded if they were not indexed in the Web of Science Core Collection; were published in a language other than English; were not primarily focused on glaucoma; were conference proceedings, abstracts, posters, editorials, or letters; or had not received citations. Full texts of eligible articles were screened using the same criteria. Disagreements were resolved by the senior author, and title and abstract screening disputes were settled by a fifth reviewer. A comprehensive search of the Web of Science database identified 1254 records. Records were ranked in descending order according to total citation count, and the 200 most-cited records were selected for title and abstract screening. Following title and abstract screening, 26 articles were excluded because they did not meet the predefined eligibility criteria, leaving 174 articles for full-text eligibility assessment. After full-text screening, 57 additional articles were excluded, leaving 117 eligible articles. These eligible articles were then ranked according to total citation count, and the 50 highest-cited articles were included in the final bibliometric analysis. The remaining 67 eligible articles were excluded because they did not rank among the Top 50 most-cited articles. The study-selection process is illustrated in (Fig. 1).

### 2.3. Data extraction and management

Four independent reviewers extracted data from each included article using a standardized form to ensure consistency. Extracted variables included first author name, article type, year of publication, total citation count, and average citations per year. To avoid overrepresentation of old publications and normalize citation accumulation across different publication years, citation density (average citation/yr) was calculated by dividing the total number of citations by the number of years since publication, Citation counts were retrieved on July 5, 2025. Additional extracted data included journal name, journal impact factor, country or region of origin, publishing institution, level of evidence, study focus, and the main findings. Any disagreements during the data extraction process were resolved through consensus among the reviewers or by consultation with a fifth reviewer. The senior author independently reviewed and verified study-design classification, evidence-level assignment, and thematic categorization to ensure methodological consistency. The level of evidence for each included study was classified according to a modified, design-based adaptation of the Oxford Centre for Evidence-Based Medicine (OCEBM) hierarchy, with Level I representing randomized controlled trials (RCTs); Level II, prospective and retrospective cohort studies; Level III, case-control studies; Level IV, case series, case reports, and cross-sectional studies; and Level V, narrative reviews or expert-opinion articles.

### 2.4. Statistical analysis

The features of the included studies were summed up using descriptive statistics. For continuous variables such as the mean age of studies and the total number of citations, this entailed determining the mean, median, and range. Frequency distributions were used to summarize categorical variables, such as the main study outcomes and the field of study. To find trends in the impact of research over time, the average number of citations was also examined. No formal risk-of-bias assessment was conducted because this bibliometric analysis aimed to characterize citation patterns, study designs, evidence levels, and thematic trends rather than to synthesize intervention effects.

## 3. Results

The bibliometric analysis of the Top 50 corpus showed a broad distribution of scholarly impact across the included articles. The corpus accumulated a total of 4054 citations, with a mean of 81.1 citations per article and a median of 67. The h-index within the Top 50 set was 45. Citation concentration analysis indicated that the top 10 articles accounted for 37.6% of total citations, the second decile contributed 21.1%, and the remaining 30 articles accounted for 41.3%. The calculated Gini coefficient was 0.26. Citation decile ranges demonstrated that articles ranked 1 to 10 received between 243 and 109 citations, while those ranked 11 to 20 received between 101 and 75 citations. Citation density, calculated by dividing the total number of citations by the number of years since publication, was used to normalize citation counts according to publication age and enable a fair comparison of scientific impact between older and more recently published studies.

Citation density ranged from 1.13 to 11.05 citations per year. The 3 most-cited articles accumulated 243, 193, and 168 citations, respectively. The most highly cited article accumulated 243 citations and had the highest citation density of 11.05 citations per year. The study evaluating the XEN45 implant had the second-highest citation density at 10.71 citations per year. Overall, although older articles accumulated the highest total citation counts, some more recently published studies demonstrated higher annual citation rates, indicating greater contemporary scientific impact. Comparison of citation density according to the level of evidence showed a slightly higher mean annual citation rate for Level I studies (3.86 citations/yr) than for Level IV studies (3.79 citations/yr); however, the difference was minimal. Citation velocity, measured as citations per year, varied across both older and more recent publications. Articles addressing 24-hour intraocular pressure monitoring had a citation density of 11.05 citations per year, while studies evaluating XEN45 implant outcomes had a citation density of 10.71 citations per year. The Top 50 most-cited glaucoma articles are summarized in Table 1.^[6-55]^

**Table 1:** Top 50 most-cited glaucoma articles.

Rank	Title	First author	Year	Complete number of citations	Citation density (citations/yr)
1	24-Hour monitoring of intraocular pressure in glaucoma management: a retrospective review^[6]^	Hughes E	2003	243	11.05
2	Calcium channel blockers in the management of low-tension and open-angle glaucoma^[7]^	Netland PA	1993	193	6.03
3	Early trabeculectomy versus conventional management in primary open angle glaucoma^[8]^	Jay JL	1988	168	4.54
4	Ophthalmic laser microendoscope ciliary process ablation in the management of neovascular glaucoma^[9]^	Uram M	1992	159	4.81
5	A long-term clinical trial of timolol therapy versus no treatment in the management of glaucoma suspects^[10]^	Epstein DL	1989	141	3.91
6	Long tube implants in management of glaucoma^[11]^	Molteno ACB	1976	140	2.85
7	The benefit of early trabeculectomy versus conventional management in primary open angle glaucoma relative to severity of disease^[12]^	Jay JL	1989	137	3.80
8	Incidence and management of glaucoma after intravitreal silicone oil injection for complicated retinal detachments^[13]^	Nguyen QH	1992	122	3.70
9	Diode laser cyclophotocoagulation – role in the management of refractory pediatric glaucomas^[14]^	Kirwan JF	2002	111	4.83
10	Clinical significance of central corneal thickness in the management of glaucoma^[15]^	Shih CY	2004	109	5.19
11	Management of uveitic glaucoma with Ahmed glaucoma valve implantation^[16]^	Da Mata A	1999	101	3.88
12	Management of acute glaucoma with pilocarpine-soaked hydrophilic lens^[17]^	Hillman JS	1974	98	1.92
13	Endocyclophotocoagulation for management of difficult pediatric glaucomas^[18]^	Neely DE	2001	91	3.79
14	Canadian perspectives in glaucoma management: setting target intraocular pressure range^[19]^	Damji KF	2003	86	3.91
15	Malignant glaucoma and its management^[20]^	Ruben S	1997	84	3.00
16	Outcomes of different management options for malignant glaucoma: a retrospective study^[21]^	Debrouwere V	2012	84	6.46
17	Analysis of surgical and medical-management of glaucoma in Sturge-Weber syndrome^[22]^	Iwach AG	1990	82	2.34
18	Pars plana vitrectomy in the management of phakic and pseudophakic malignant glaucoma^[23]^	Harbour JW	1996	79	2.72
19	Glaucoma management among individuals enrolled in a single comprehensive insurance plan^[24]^	Friedman DS	2005	75	3.75
20	One-year result of XEN45 implant for glaucoma: efficacy, safety, and postoperative management^[25]^	Tan SZ	2018	75	10.71
21	Comparison of mitomycin C trabeculectomy, glaucoma drainage device implantation, and laser neodymium:YAG cyclophotocoagulation in the management of intractable glaucoma after penetrating keratoplasty^[26]^	Ayyala RS	1998	74	2.74
22	Surgical management of chronic glaucoma in aphakia^[27]^	Bellows AR	1983	69	1.64
23	Surgical management of secondary glaucoma after pars plana vitrectomy and silicone oil injection for complex retinal detachment^[28]^	Budenz DL	2001	69	2.88
24	Removal of silicone oil in the management of glaucoma in eyes with emulsified silicone^[29]^	Moisseiev J	1993	67	2.09
25	Management of bleb leaks after glaucoma filtering surgery – use of autologous fibrin tissue glue as an alternative^[30]^	Asrani SG	1996	67	2.31
26	A randomised, prospective study comparing trabeculectomy augmented with antimetabolites with a viscocanalostomy technique for the management of open angle glaucoma uncontrolled by medical therapy^[31]^	O’Brart DPS	2002	67	2.91
27	New surgical approach in the management of pseudophakic malignant glaucoma^[32]^	Lois N	2001	66	2.75
28	Incidence and management of encapsulated cysts following Ahmed glaucoma valve insertion^[33]^	Eibschitz-Tsimhoni M	2005	64	3.20
29	Baerveldt glaucoma implant in the management of refractory childhood glaucomas^[34]^	Budenz DL	2004	63	3.00
30	Comparison of the ocular hypotensive lipid AGN 192024 with timolol – dosing, efficacy and safety evaluation of a novel compound for glaucoma management^[35]^	Laibovitz RA	2001	62	2.58
31	Endoscopic diode laser cyclophotocoagulation in the management of aphakic and pseudophakic glaucoma in children^[36]^	Carter BC	2007	61	3.39
32	Long-term effects of tube-shunt procedures on management of refractory childhood glaucoma^[37]^	Eid TE	1997	58	2.07
33	Management of glaucoma in patients with nanophthalmos^[38]^	Yalvac IS	2008	58	3.41
34	Post-penetrating keratoplasty glaucoma: risk factors, management and visual outcome^[39]^	Sihota R	1998	57	2.11
35	Direct costs of glaucoma management following initiation of medical therapy – a simulation model based on an observational study of glaucoma treatment in Germany^[40]^	Kobelt G	1998	54	2.00
36	Assessment of patient opinions of different clinical tests used in the management of glaucoma^[41]^	Gardiner SK	2008	54	3.18
37	Service innovation in glaucoma management: using a web-based electronic patient record to facilitate virtual specialist supervision of a shared care glaucoma programme^[42]^	Wright HR	2015	54	5.40
38	Management of chronic or intermittent primary angle-closure glaucoma: a long-term follow-up of the results of peripheral iridectomy used as an initial procedure^[43]^	Playfair TJ	1979	52	1.13
39	Management of glaucoma in pregnancy and lactation^[44]^	Johnson SM	2001	52	2.17
40	A randomised, prospective study comparing trabeculectomy with viscocanalostomy with adjunctive antimetabolite usage for the management of open angle glaucoma uncontrolled by medical therapy^[45]^	O’Brart DPS	2004	52	2.48
41	Selective laser trabeculoplasty for the management of open-angle glaucoma in St. Lucia^[46]^	Realini T	2013	52	4.33
42	Endoscopic cyclophotocoagulation (ECP) in the management of uncontrolled glaucoma with prior aqueous tube shunt^[47]^	Francis BA	2011	51	3.64
43	Anti-VEGF treatment is the key strategy for neovascular glaucoma management in the short term^[48]^	Sun YY	2016	49	5.44
44	Glaucoma in penetrating keratoplasty: risk factors, management and outcome^[49]^	Huber KK	2013	46	3.83
45	Patient persistency with pharmacotherapy in the management of glaucoma^[50]^	Reardon G	2003	45	2.05
46	A pilot study of lens extraction in the management of acute primary angle-closure glaucoma^[51]^	Zhi ZM	2003	45	2.05
47	Sequential office pressure measurements in the management of glaucoma^[52]^	Collaer N	2005	45	2.25
48	Lensectomy in the management of glaucoma in spherophakia^[53]^	Willoughby CE	2002	42	1.83
49	Comparison of machine-learning classification models for glaucoma management^[54]^	An GZ	2018	41	5.86
50	Agreement between specially trained and accredited optometrists and glaucoma specialist consultant ophthalmologists in their management of glaucoma patients^[55]^	Marks JR	2012	40	3.08

Citation density was calculated by dividing the total number of citations by the number of years since publication, using July 5, 2025 as the citation cutoff year.

### 3.1. Decadal distribution

**Figure 2.**
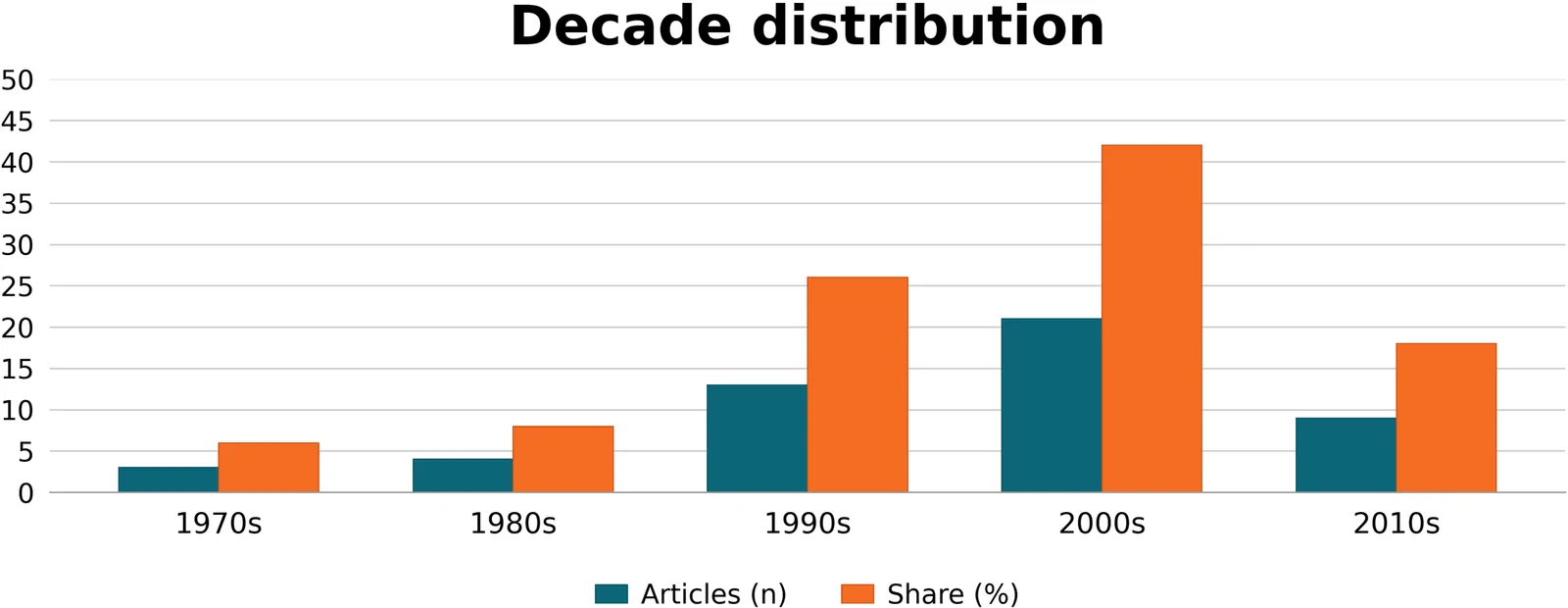
Decadal distribution of the Top 50 most-cited glaucoma articles, showing the number of articles and percentage contribution by decade.

The temporal distribution of the top 50 articles spanned 5 decades, from 1974 to 2018. The highest concentration of publications occurred in the 2000s, which contributed 21 articles (42.0%), followed by the 1990s with 13 articles (26.0%). Together, these 2 decades accounted for 34 articles (68.0%) of the top 50 corpus. The 2010s contributed 9 articles (18.0%). Earlier publications were less frequent, with 4 articles (8.0%) published in the 1980s and 3 articles (6.0%) in the 1970s. Overall, 43 articles (86.0%) were published between 1990 and 2019. The decadal distribution is illustrated in Figure 2 and presented in Table 2.

**Table 2:** Decadal distribution of the top 50 most-cited glaucoma articles.

Decade	Year range	Articles (n)	Share (%)
1970s	1970–1979	3	6.0
1980s	1980–1989	4	8.0
1990s	1990–1999	13	26.0
2000s	2000–2009	21	42.0
2021s	2010–2019	9	18.0

### 3.2. Journal distribution (with JCR 2024)

The top 50 corpus demonstrated a clear concentration in a relatively small group of ophthalmology journals. OPHTHALMOLOGY accounted for the highest output, with 15 articles and 1311 citations, followed by the BRITISH JOURNAL OF OPHTHALMOLOGY with 7 articles and 575 citations. The JOURNAL OF GLAUCOMA and EYE each published 4 articles, accumulating 403 and 310 citations, respectively. ARCHIVES OF OPHTHALMOLOGY and GRAEFE’S ARCHIVE FOR CLINICAL AND EXPERIMENTAL OPHTHALMOLOGY each contributed 3 articles, with 250 and 184 citations, respectively. The AMERICAN JOURNAL OF OPHTHALMOLOGY and JOURNAL OF AAPOS each published 2 articles, accumulating 238 and 152 citations, respectively. The SOUTH AFRICAN MEDICAL JOURNAL and CANADIAN JOURNAL OF OPHTHALMOLOGY each contributed 1 article, with 140 and 86 citations, respectively. The remaining 8 articles were distributed across 8 additional journals and accounted for 405 citations. Overall, the 8 journals contributing at least 2 articles accounted for 40 of the 50 articles (80.0%) and 3423 of 4054 citations (84.4%). Journal distribution of the Top 50 most-cited glaucoma articles is detailed in Table 3.

**Table 3:** Top journals contributing to the top 50 most-cited glaucoma articles.

Rank	Journal	Number of papers	Total citations	JCR impact factor (2024)
1	Ophthalmology	15	1311	~9.5
2	British Journal of Ophthalmology	7	575	3.5
3	Journal of Glaucoma	4	403	–
4	Eye	4	310	3.2
5	Archives of Ophthalmology	3	250	–
6	Graefe’s Archive for Clinical & Experimental Ophthalmology	3	184	~2.3
7	American Journal of Ophthalmology	2	238	~4.2
8	Journal of AAPOS	2	152	–
9	South African Medical Journal	1	140	–
10	Canadian Journal of Ophthalmology	1	86	–
–	Other journals (n = 8)	8	Remaining citations	–

JCR = Journal Citation Reports.

Additional publications were identified in ARCHIVES OF OPHTHALMOLOGY and the AMERICAN JOURNAL OF OPHTHALMOLOGY (2 papers each; 171 and 238 citations, respectively), providing historical and translational perspectives. Less frequent but still notable venues included JAMA OPHTHALMOLOGY, BMC OPHTHALMOLOGY, and the JOURNAL OF CATARACT AND REFRACTIVE SURGERY (all single-paper placements), indicating that well-placed contributions in broad or adjacent ophthalmic outlets can yield meaningful impact. Overall, the top 8 journals (≥2 articles each) accounted for 36/50 articles (72.0%) and nearly 3-quarters of the total citation load, while the remaining journals formed a long tail of 14 papers with dispersed influence.

### 3.3. Research setting and geographic distribution

**Figure 3.**
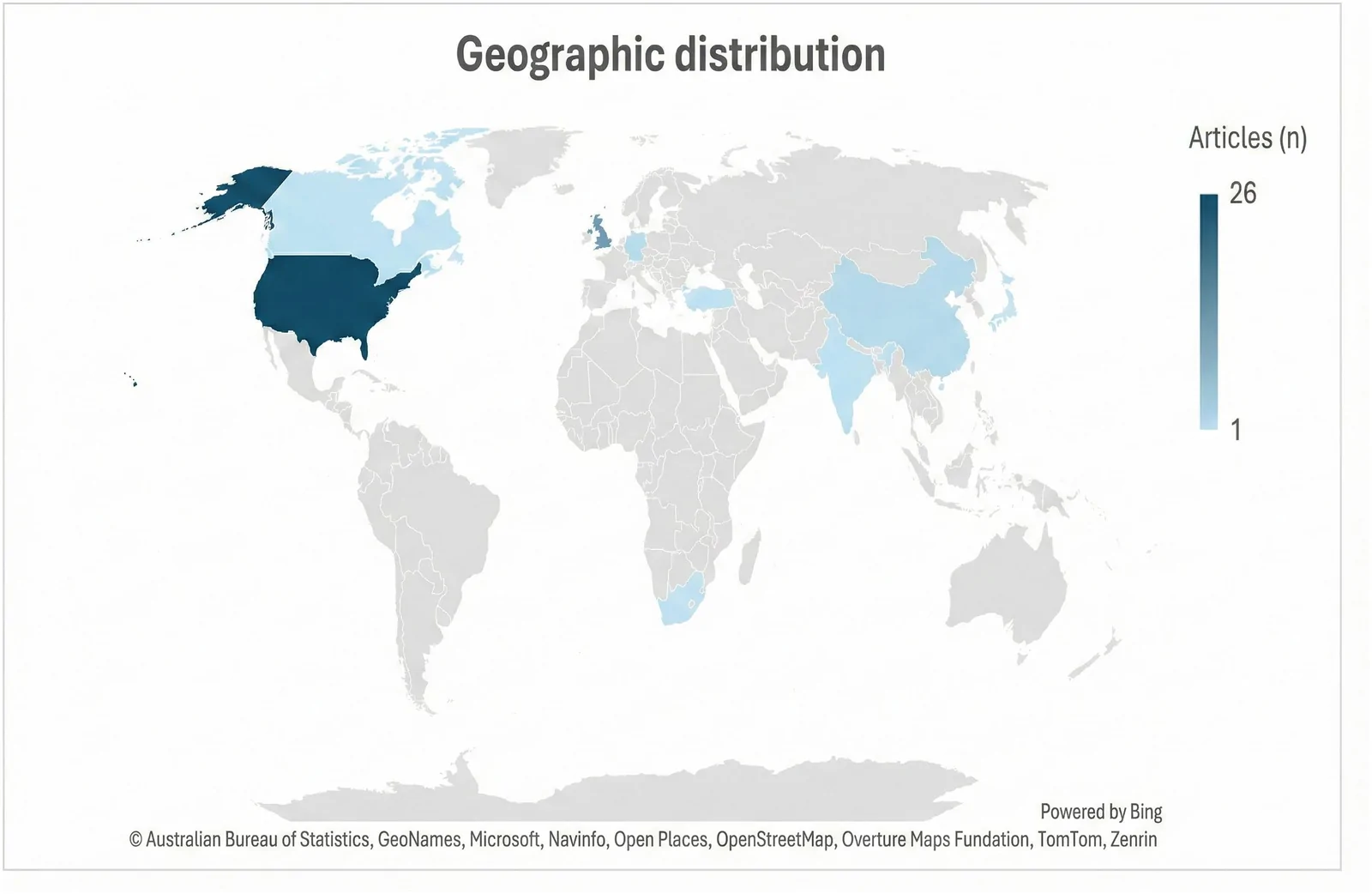
Geographic distribution of the Top 50 most-cited glaucoma articles by country of origin.

Analysis of the Top 50 glaucoma corpus showed a concentration of publications originating from North America and Western Europe. The geographic distribution of the Top 50 most-cited glaucoma articles by country is shown in Figure 3 and Table 4. The United States accounted for more than half of the output (26/50; 52.0%). The United Kingdom, listed under multiple variants in the dataset, contributed a combined total of 15 articles (30.0%).

**Table 4:** Geographic distribution of the top 50 most-cited glaucoma articles by country.

Country	Articles (n)	Share (%)
United States	26	52.0
United Kingdom*	15	30.0
China	2	4.0
Germany	2	4.0
Canada	1	2.0
India	1	2.0
Japan	1	2.0
South Africa	1	2.0
Turkey	1	2.0

* The United Kingdom includes multiple country name variants in the database.

Asian countries contributed a smaller proportion of publications, including China (2; 4.0%), Japan (1; 2.0%), and India (1; 2.0%). Additional contributions were identified from Canada (1; 2.0%), Germany (2; 4.0%), South Africa (1; 2.0%), and Turkey (1; 2.0%).

Collectively, these data demonstrate the geographic distribution of the Top 50 most-cited glaucoma articles across multiple regions.

### 3.4. Evidence hierarchy of the 50 most-cited glaucoma articles

The evidence profile of the corpus showed a predominance of cohort-based and descriptive study designs. Levels of evidence among the Top 50 most-cited glaucoma articles are summarized in Table 5. Level II evidence, comprising retrospective and prospective cohort studies, was the most common category (22/50, 44.0%). Level IV evidence, comprising case series, case reports, and cross-sectional studies, accounted for 19 articles (38.0%). Level I evidence, represented by RCTs, accounted for 6 articles (12.0%). Level V evidence, including narrative reviews and expert-opinion articles, accounted for 3 articles (6.0%). No Level III case-control studies were identified.

**Table 5:** Level of evidence among the top 50 most-cited glaucoma articles.

Level of evidence	Articles (n = 50)	Percentage (%)
Level I – randomized controlled trials	6	12
Level II – cohort study (Prospective/Retrospective)	22	44
Level III – case-control study	0	0
Level IV – case series, case reports, and cross-sectional studies	19	38
Level V – expert opinion/narrative review	3	6

### 3.5. Study-design distribution of the top 50 most-cited glaucoma articles

Study-design distribution of the Top 50 glaucoma articles is presented in Table 6. Retrospective cohort studies comprised the largest category (18/50; 36.0%). Case series accounted for 15/50 (30.0%). RCTs contributed 6/50 (12.0%), while prospective cohort studies accounted for 4/50 (8.0%). Cross-sectional studies and narrative reviews contributed equally, as each design represented 3/50 (6.0%). Case reports accounted for 1/50 (2.0%). Overall, the distribution demonstrates a predominance of retrospective and case series-based study designs among the Top 50 most-cited glaucoma articles.

**Table 6:** Study-design distribution of the top 50 most-cited glaucoma articles.

Study design	Number of papers	%
Retrospective cohort study	18	36
Case series	15	30
Randomized controlled trial	6	12
Prospective cohort study	4	8
Cross-sectional study	3	6.0
Narrative review/expert opinion	3	6
Case report	1	2.0
Total	50	100.0

**Figure 4.**
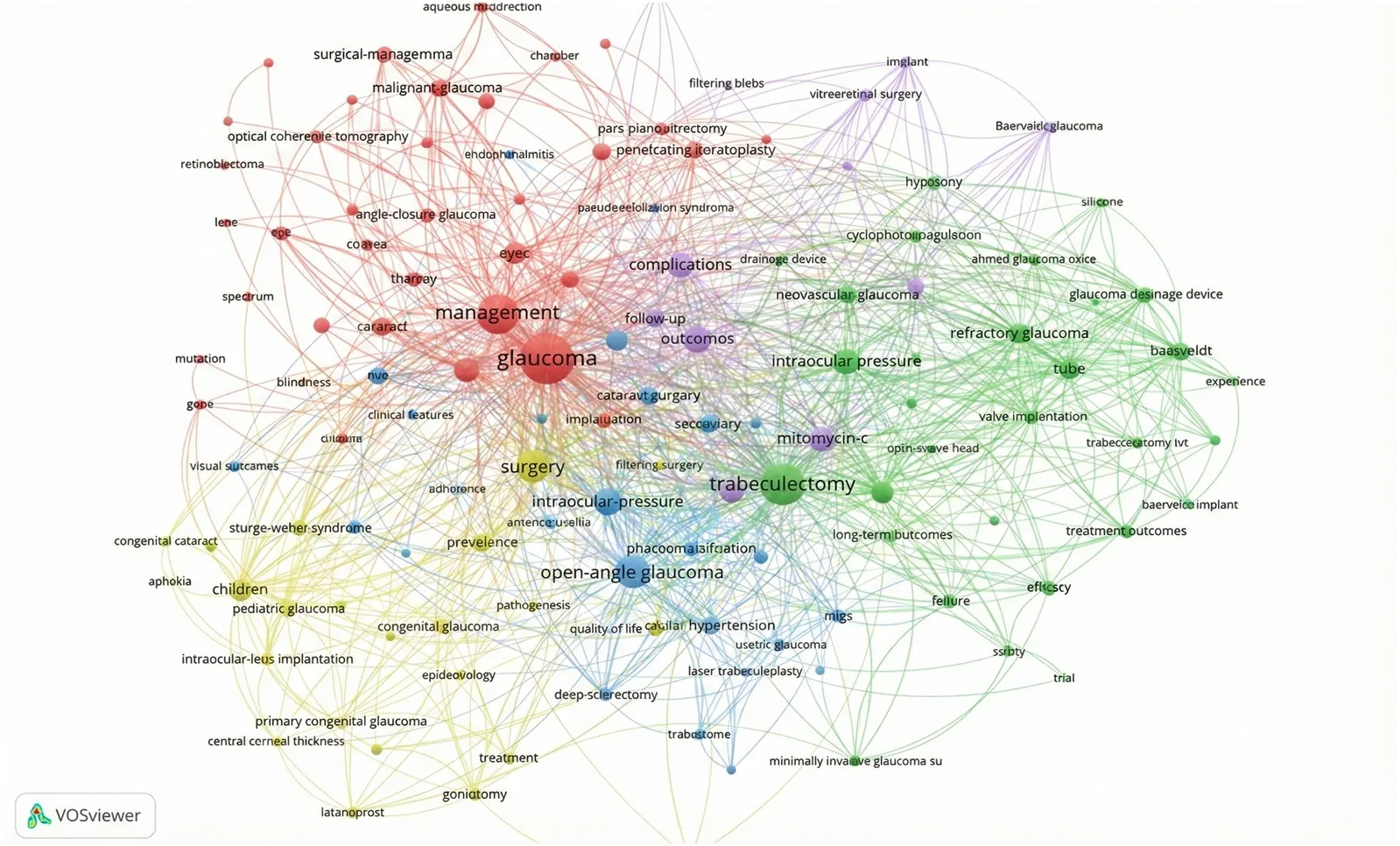
Keyword co-occurrence network of the Top 50 most-cited glaucoma articles generated using VOSviewer, illustrating major thematic clusters within the glaucoma literature.

The keyword co-occurrence map generated through VOSviewer is presented in Figure 4 and shows a clustered thematic structure within the Top 50 glaucoma literature, describing how research topics are distributed across interrelated domains.^[56]^ The central red cluster is anchored by the keywords “glaucoma” and “management” and is linked with terms such as angle-closure glaucoma, malignant glaucoma, cataract, optical coherence tomography, and endophthalmitis. This cluster represents clinical management themes and commonly associated comorbid conditions. The green cluster includes surgical-related terms, including trabeculectomy, IOP, mitomycin-C, Baerveldt implant, Ahmed valve, and glaucoma drainage devices. These keywords represent pressure-lowering surgical approaches and adjunctive antifibrotic strategies.

A yellow–blue cluster connects open-angle glaucoma, surgery, ocular hypertension, phacoemulsification, and MIGS. This cluster corresponds to newer surgical modalities, perioperative management, and population-based perspectives.

The purple cluster includes terms such as cyclophotocoagulation, hypotony, tube devices, and refractory glaucoma, representing complication management and advanced device-based interventions.

Overall, the map shows that the high-impact glaucoma literature is organized into multiple interconnected thematic areas rather than a single dominant focus. These include diagnostic and management integration, conventional filtration surgery and adjunct therapies, newer surgical innovations such as MIGS, and management of complications and refractory disease. The presence of linkages between clusters indicates frequent co-occurrence of topics across studies, suggesting conceptual connectivity among surgical techniques, devices, and outcome-focused research areas.

## 4. Discussion

This bibliometric analysis of the Top 50 most-cited articles in glaucoma management demonstrates that scholarly influence is broadly distributed rather than concentrated in a few seminal publications. The citation profile showed a balanced pattern across decades and study designs, supported by a moderate Gini coefficient.

Earlier bibliometric studies have described glaucoma research from a broad field perspective. Frings et al examined the 100 most-cited glaucoma articles and reported that clinical papers were mainly concerned with diagnostics and epidemiology, alongside basic research.^[57]^ Huang et al mapped 6427 PubMed-indexed glaucoma publications and identified a broad range of themes, including diagnostic testing, medical therapy, surgery, genetics, pathology, and epidemiology.^[58]^

The present study complements rather than replaces these broader analyses. By examining an updated, citation-ranked corpus retrieved using a glaucoma-management search strategy, it describes the distribution of scholarly attention across diagnostic assessment, conventional filtration surgery, MIGS, tube devices, and complication management. Its findings should therefore be interpreted as a citation-based characterization of the Top 50 corpus, rather than as a comprehensive account of all glaucoma research or as direct guidance for patient-specific treatment decisions.

The persistence of highly cited articles from earlier and recent decades highlights the durability of key contributions in glaucoma care. Foundational studies, such as Molteno’s work on long-tube implants, established the basis for modern glaucoma drainage surgery and remain relevant.^[11]^ Similarly, Jay’s trials comparing early trabeculectomy with medical management continue to guide surgical decisions, particularly by disease severity.^[8,12]^ More recent studies have also achieved rapid citation uptake. Hughes’ work on 24-hour IOP monitoring provided key insights into diurnal fluctuations and remains the most-cited article in this corpus.^[6]^ Likewise, Tan’s evaluation of the XEN45 gel stent marked an important milestone in MIGS, supporting the safety and efficacy of device-based interventions.^[25]^

The coexistence of classical and emerging surgical techniques reflects the evolutionary nature of glaucoma management. Traditional approaches such as trabeculectomy and tube shunts remain central due to their proven long-term outcomes.^[8,11,12]^ Meanwhile, innovations like endocyclophotocoagulation and MIGS have expanded treatment options, particularly for refractory or moderate disease.^[18,47]^ Within the Top 50 highly cited literature, this pattern suggests a gradual integration rather than replacement, reflecting an evolving continuum of care.

Geographically, high-impact research is concentrated in the United States and the United Kingdom, likely reflecting strong academic and research infrastructure. However, increasing contributions from Asia, particularly China, Japan, and India, indicate growing global diversification. Journal analysis shows that Ophthalmology and the British Journal of Ophthalmology are primary publication platforms, with subspecialty journals such as the Journal of Glaucoma playing a complementary role.

Study design analysis revealed a predominance of retrospective cohorts and case series, reflecting challenges in conducting randomized surgical trials, including ethical constraints and variability in technique. Nevertheless, the proportion of RCTs exceeds that of many surgical fields, likely due to the use of IOP as a measurable endpoint. Trials such as those by O’Brart and colleagues demonstrate rigorous experimental design in ophthalmic surgery.^[31,45]^

Thematic analysis showed considerable diversity, including surgical techniques, diagnostics, complications, and disease subtypes. Topics such as malignant glaucoma, hypotony, and refractory disease appear alongside studies of trabeculectomy, drainage devices, and MIGS, highlighting the complexity and integrated nature of glaucoma management.^[20,21]^

Recent trends point to a growing focus on MIGS, reflecting interest in approaches that reduce surgical morbidity without compromising IOP control. The expanding evidence base for MIGS devices, together with continued refinement of established procedures such as trabeculectomy and tube shunt surgery, suggests that surgical management is moving toward a broader range of options tailored to disease severity and individual patient risk. This trend is likely to continue as longer-term outcome data for newer devices accumulate, allowing more precise positioning of MIGS within the surgical algorithm. This study has several strengths, including the use of the Web of Science database for standardized indexing, strict inclusion criteria, and independent data extraction to reduce bias. Bibliometric indicators provided a clear overview of citation patterns, geographic trends, and study designs.

Several limitations should be acknowledged. First, citation analyses inherently favor older publications, high-impact journals, and research from well-resourced regions, regardless of actual clinical merit. Second, restricting our search to the Web of Science and English-language articles likely excluded relevant global literature. PubMed indexing was not assessed or used as an eligibility criterion. Furthermore, using a Top 50 cutoff may exclude studies of considerable clinical relevance that have not yet accumulated comparable citations.

More fundamentally, citation count primarily reflects a publication’s visibility and historical position within the field, not the rigor of its methodology or its level of evidence. Many early papers accrue citations merely by being first, often serving as background context rather than the basis for current clinical decisions. Citation count is not a direct measure of clinical effectiveness. In fact, recent randomized trials from less prominent regions might carry significantly more weight in daily practice despite having a modest bibliometric profile.

Our use of VOSviewer to map keyword co-occurrence and thematic clustering offers a useful view of the conceptual structure within the glaucoma literature, though the analysis remains descriptive rather than relational in scope. A dataset of 50 articles is too small to support institutional collaboration networks, citation-burst detection, or author productivity mapping with any statistical reliability, as these methods depend on substantially larger corpora to produce stable, interpretable results. Such approaches are better suited to bibliometric studies spanning hundreds or thousands of records, where collaboration patterns and citation trajectories can be tracked with confidence over time. Therefore, our findings describe patterns of scholarly attention, not a hierarchy of evidence. Treatment decisions must continue to be guided by systematic reviews, clinical practice guidelines, and rigorous evidence appraisal rather than citation metrics alone.

## 5. Conclusion

This bibliometric analysis of the Top 50 most-cited glaucoma-management articles identified a corpus dominated by retrospective cohort and case-series designs, with contributions concentrated in the United States and the United Kingdom. Highly cited literature spanned diagnostic assessment, traditional filtration surgery, MIGS and device-based approaches, and management of complications. These findings should be interpreted as citation-based patterns within this selected corpus, rather than as estimates of the entire evidence base, comparative effectiveness, or future direction of glaucoma care. Future research should continue to evaluate emerging approaches through rigorous comparative studies and broader international representation.

## Acknowledgments

The authors would like to thank Research Hive for providing research training and statistical guidance. The authors acknowledge the use of ChatGPT (OpenAI) for language editing, grammar correction, and wording refinement. All final content was reviewed, verified, and approved by the authors. ChatGPT was not used for data collection, data analysis, citation generation, or the production of figures, tables, or graphical elements.

## Author contributions

**Data curation:** Beta Abuhadi, Jumanah M. Aldirbas.

**Project administration:** Beta Abuhadi, Fai H. Alotaibi.

**Investigation:** Naya F. Alzahrani, Fatema M. Almulhim, Jumana I. Alhashmi Alamir, Lina Justenia, Razan A. Abuhekmah, Fatima A. Almadhloum Alsuwaidi, Rawan A. Abuhekmah.

**Methodology:** Najla A. Alsubaie, Abdulwahab M. Alqaffas.

**Supervision:** Fahad AlHarthi.

**Validation:** Fahad AlHarthi.

**Visualization:** Beta Abuhadi.

**Writing – review & editing:** Beta Abuhadi, Jumanah M. Aldirbas, Fatema M. Almulhim, Jumana I. Alhashmi Alamir, Lina Justenia, Fatima A. Almadhloum Alsuwaidi.

**Writing – original draft:** Naya F. Alzahrani, Fai H. Alotaibi, Najla A. Alsubaie, Razan A. Abuhekmah, Fatima A. Almadhloum Alsuwaidi, Abdulwahab M. Alqaffas, Rawan A. Abuhekmah.
